# Benefits and side effects of protein supplementation and exercise in sarcopenic obesity: A scoping review

**DOI:** 10.1186/s12937-023-00880-7

**Published:** 2023-10-23

**Authors:** Khang Jin Cheah, Lin Jia Cheah

**Affiliations:** 1 Department of Allied Health Sciences, Faculty of Science, Universiti Tunku Abdul Rahman, Jalan Universiti, Bandar Barat, Kampar, 31900 Malaysia; 2 Centre for Biomedical and Nutrition Research, Universiti Tunku Abdul Rahman, Jalan Universiti, Bandar Barat, Kampar, 31900 Malaysia; 3 Department of Health, North West Regional Hospital, Cooee, Tasmania 7320 Australia

**Keywords:** Sarcopenia, Obesity, Sarcopenic obesity, Protein supplement, Whey protein, Exercise

## Abstract

**Background:**

Protein supplements have been widely used among those who are struggling with sarcopenic obesity among older adults. However, despite their popularity, there is still a lack of concrete evidence on both the potential benefits and side effects of protein supplementation and exercise on sarcopenic obesity (SO).

**Objective:**

Thus, we aimed to determine the impacts of protein supplementation and exercise in older adults with sarcopenic obesity.

**Method:**

A systematic database search was conducted for randomised controlled trials, quasi experimental study and pre-post study design addressing the effects of protein supplementation in improving sarcopenic obesity among older adults. This scoping review was conducted based on PRISMA-Scr guidelines across PubMed, Embase, Web of Science and Cochrane Library databases. To assess record eligibility, two independent reviewers performed a rigorous systematic screening process.

**Results:**

Of the 1,811 citations identified, 7 papers met the inclusion criteria. Six studies were randomised controlled trials and one study was a pre-post test study design. The majority of studies discussed the use of both protein supplements and exercise training. The included studies prescribed protein intake ranging from 1.0 to 1.8 g/kg/BW/day for the intervention group, while the duration of exercise performed ranged from 2 to 3 times per week, with each session lasting for 1 hour. Whey protein supplementation has been shown to be effective in improving sarcopenic conditions and weight status in SO individuals. The combination of exercise training especially resistance training and the used of protein supplement provided additional benefits in terms of lean muscle mass as well as biomarkers. The study also revealed a lack of consistency in exercise design among interventions for sarcopenic obesity.

**Conclusion:**

Overall, it appears to be a promising option for SO individuals to improve their sarcopenic condition and weight status through the combination of resistance exercise and whey protein supplementation. However, it also highlights the need for caution when it comes to high amounts of protein intake prescription. Future research is warranted to investigate the optimal exercise design for this population, given the limited research conducted in this specific area.

## Introduction

With the rapid growing of older adult population worldwide, age-related health problems have become a global concern. According to the United Nations, the global population aged 60 years or over numbered 962 million in 2017, which is approximately 13% of the global population [1]. The World Health Organization (WHO) reported that the number had increased to one billion in 2020, outnumbering children younger than five years old. [2]. This figure is anticipated to double by 2050, reaching 2.1 billion [2]. The issue of ageing population is not only limited to high-income countries. In low- and middle-income countries, the number of people aged 60 years and over is growing faster than in high-income countries [3]. The pace of population ageing is much faster than in the past, as a result it is estimated that 80% of older people will be living in low- and middle-income countries [2].

Research has shown that older adults are at a considerably higher risk of health problems [4] including sarcopenia [5–7] and sarcopenic obesity [8, 9]. Sarcopenia is a condition that affects older people more frequently than younger people, with prevalence rates ranging from 5 to 50% [5] depending on diagnostic criteria and geographic location. For instance, among Asian countries, Thailand, Malaysia and Singapore showed prevalence rates of sarcopenia of 22.2%, 59.8% and 32.2%, respectively [7, 10, 11]. As a high-risk geriatric syndrome, by using different definitions, a study conducted in Canada reported prevalence of sarcopenic obesity ranging from 0.1 to 85.3% in males, and from 0 to 80.4% in females [9]. It is noted that the prevalence of sarcopenic obesity is increasing in adults aged 65 years and older [8] and it appears to be particularly common among older women [12].

Ageing people may experience change in visceral fat distribution into the intra-abdominal region due to adipose inflammation, which can also promote fat infiltration inside the skeletal muscles, ultimately resulting in loss of overall strength and functional ability [13]. This disease is referred to as sarcopenia, which is the loss of muscle mass that occurs with ageing and is significantly linked to an increased risk of injury occurrences [14], poor mental health, cognitive decline, decreased physical activity [15] and overall increased mortality [14, 16]. On the other hand, sarcopenic obesity refers to the combination of sarcopenia and obesity [17]. Sarcopenia can occur in obese individuals at any age as a result of the detrimental effects of adipose tissue-dependent metabolic abnormalities, such as oxidative stress, inflammation, and insulin resistance, all of which have a significant negative impact on muscle mass [18].

Sarcopenia and obesity are considered as double health burden as it can independently pose increased risks for adverse health outcomes. For example, individuals with obesity have a high prevalence of chronic non-communicable diseases that negatively impact muscle metabolism [19, 20]. Compared to individuals who only have sarcopenia or obesity, individuals with sarcopenic obesity have greater risks of metabolic disorders, higher CVD prevalence, higher mortality rates and reduced physical performance [21–23]. The health hazards may be increased synergistically when these two disorders are present [8, 23]. Moreover, individuals with sarcopenic obesity have a higher risk of developing chronic conditions such as systemic inflammation, full-blown sarcopenia, cachexia as well as systemic insulin resistance and other related clinical issues [13].

In 2015, a study was conducted to investigate the effects of a high whey protein, leucine and vitamin D-enriched supplement on muscle mass during intentional weight loss among obese older adults. The outcomes demonstrated that the use of the supplement was successful in maintaining muscle mass while losing weight in this cohort [24]. On the other hand, a six-month experiment among older people with sarcopenia aiming to examine the safety and tolerability of a medical nutrition drink fortified with vitamin D, calcium, and leucine revealed that the drink was safe and well-tolerated by the participants [25]. The results from these studies have suggested that the oral supplement drink containing protein supplement may have prospective advantages for the treatment of sarcopenic obese older people without compromising muscle mass and strength. These studies have highlighted the potential efficacy of protein supplementation in attenuating the negative impact of sarcopenic obesity on muscle health.

However, recent review studies have indicated that protein supplementation alone may not lead to significant changes in parameters associated with sarcopenia [26, 27], which contradicts the findings from previous studies [28]. On the other hand, a meta-analysis review has shown that exercise training alone or in combination with protein supplementation improved muscle mass, grip strength, reduced total fat mass, as well as waist circumference in individuals with sarcopenia [29]. The evidence suggests that exercise and protein supplementation have a synergistic effect on the condition of sarcopenia among individuals with sarcopenia [30, 31]. However, none of these systematic reviews specifically focused on the sarcopenic obesity population, and the potential side effects of protein supplementation were not reported. Among the previous review studies on sarcopenic obesity, greater emphasis was placed on the effects of exercise [32, 33], while the effect of protein supplementation remains relatively underreported. Taken together, the findings regarding the effects of protein supplementation on sarcopenia were inconclusive. Therefore, the aim of the present scoping review is to identify the various types of protein supplements available, assess their effects on sarcopenia and obesity, evaluate the potential side effects, and to determine the impact of exercise among older adults with sarcopenic obesity.

## Method

### Study design

We conducted a scoping review to summarise the available evidence and provide an overview of protein supplementation intervention and their outcomes related to the sarcopenia and weight status of older adults with sarcopenic obesity. The methodological framework proposed by Arksey and O’Malley [34] was used to conduct this scoping review which involved the following steps: (i) identifying the research question, (ii) identifying relevant studies, (iii) selecting the studies, (iv) charting the information and (v) summarizing the results. The present scoping review was reported in accordance with Preferred Reporting Items for Systematic Review and Meta-analysis extension for scoping review (PRISMA-ScR) guidelines [35].

### Identifying research question

This review was led by the following research questions: (i) What are the types of protein supplementation commonly used for sarcopenic obesity? (ii) What are the effects of protein supplementation intervention on sarcopenic obesity in older adults? and (iii) What are the side effects of protein supplementation?

### Identifying relevant studies

A comprehensive search was performed using the databases in PubMed, Embase, Web of Science and Cochrane Library. We searched for articles that were published between 25 December 2012 and 1 February 2023 (last 10 years). The search was limited to the last 10 + years because authors perceived those interventions earlier than 10 + years might not be as relevant in the current scenario. Additionally, an internet search was conducted using various combinations of relevant search terms in the Google search engine, reviewing the first 10 pages of search results to identify any potentially relevant articles. This review examined the effectiveness of protein supplementation, compared protein supplement with control, and provided quantitative measurements of muscle strength, body composition, or frailty. Randomised or quasi-randomised controlled trials that included adults with sarcopenic obesity were used in this review. Protein-supplemented or control groups which co-ingested other potentially anabolic agents (e.g. testosterone, creatine) were not considered. The search plan followed the following search string and key search terms used in the search for articles are as listed in Table 1.

**Table 1:** The search string and key search terms used in the study

# of Search	Search Term(s)
#1	“sarcopenic obesity” OR “sarcopenic adiposity” OR “lipotoxic sarcopenia” OR sarcopenia OR “muscle loss” OR “amyotrophy” OR “sarcobesity” OR “sarcopenic obese” OR “obese sarcopenia”
#2	“Protein” OR “Amino Acid” OR “Protein supplement” OR “Dietary protein” OR “Dietary amino acid” OR “nutritional supplement” OR “Dietary supplement” OR “Oral supplement”
#3	#1 AND #2

### Study selection

The screening process consisted of two stages: (1) a title and abstract screening and; (2) full-text screening. Dietary supplementation of protein or amino acids from all sources was considered. Sarcopenic obesity was deemed eligible if the article clearly mentioned its definition. Articles were included based on predefined PICOS (Participants, Intervention, Comparison, Outcomes, Study design) criteria. The description of the PICOS criteria used to define the research question is presented in Table 2.

**Table 2:** PICOS criteria for inclusion of studies

**Population**	Human subjects > 55 years old with sarcopenic obesity
**Intervention**	Consumption of and/or adherence to the supplement intake, different supplement dosage, exercise training
**Comparison**	Without consumption of and/or adherence of different supplement intake, supplement dosage, exercise training
**Study design**	Randomized controlled trials/Quasi-experimental/ pre-post study
**Outcome**	BIA skeletal muscle index, changes in body composition, muscle mass, fat mass, weight, BMI, waist circumference, biochemical data
**Research Question**	What is the effect of protein supplementation and exercise on body composition in sarcopenic obesity adults?

Studies meeting the following criteria were included: (1) participants aged 55 years or above; (2) healthy participants with sarcopenia, defined with at least one of the following indicators: muscle mass loss, low muscle strength, or poor physical performance; (3) the intervention group was with protein or amino acid supplementation, and the comparison group was exercise alone or with placebo supplementation; (4) study design: RCTs; and (5) outcome: muscle strength, muscle mass, and physical performance.

Main reasons for exclusion of articles from the scoping review were: (1) undefined classification of sarcopenic obesity; (2) description study, observation study, animal study; (3) clinical research with patient populations diagnosed with chronic and acute disorders or receiving treatments that may independently lead to catabolic changes in protein turnover with negative effects on skeletal muscle mass/function.

Two investigators independently read the full texts of the articles that were not excluded at the initial stage, then selected the studies that met the inclusion criteria. Any disagreements in article selection were resolved through discussion and consensus.

### Charting the data

Data charting was primarily completed by two independent reviewers (K.J. and L.J.) using a pre-established template. Data were extracted from this review based on the following categories: (a) study characteristics, (b) methodological characteristics, (c) intervention strategies, and (d) targeted outcomes.

### Collating, summarizing, and reporting the results

A thematic narrative synthesis of included articles summarising the effectiveness of each intervention strategy on sarcopenic obesity was carefully extracted from each included article.

### Ethics

Ethical approval was not required from the Medical Research and Ethics Committee as data were collected from existing publications (i.e., secondary data) and no humans were directly contacted.

## Results

**Fig. 1:**
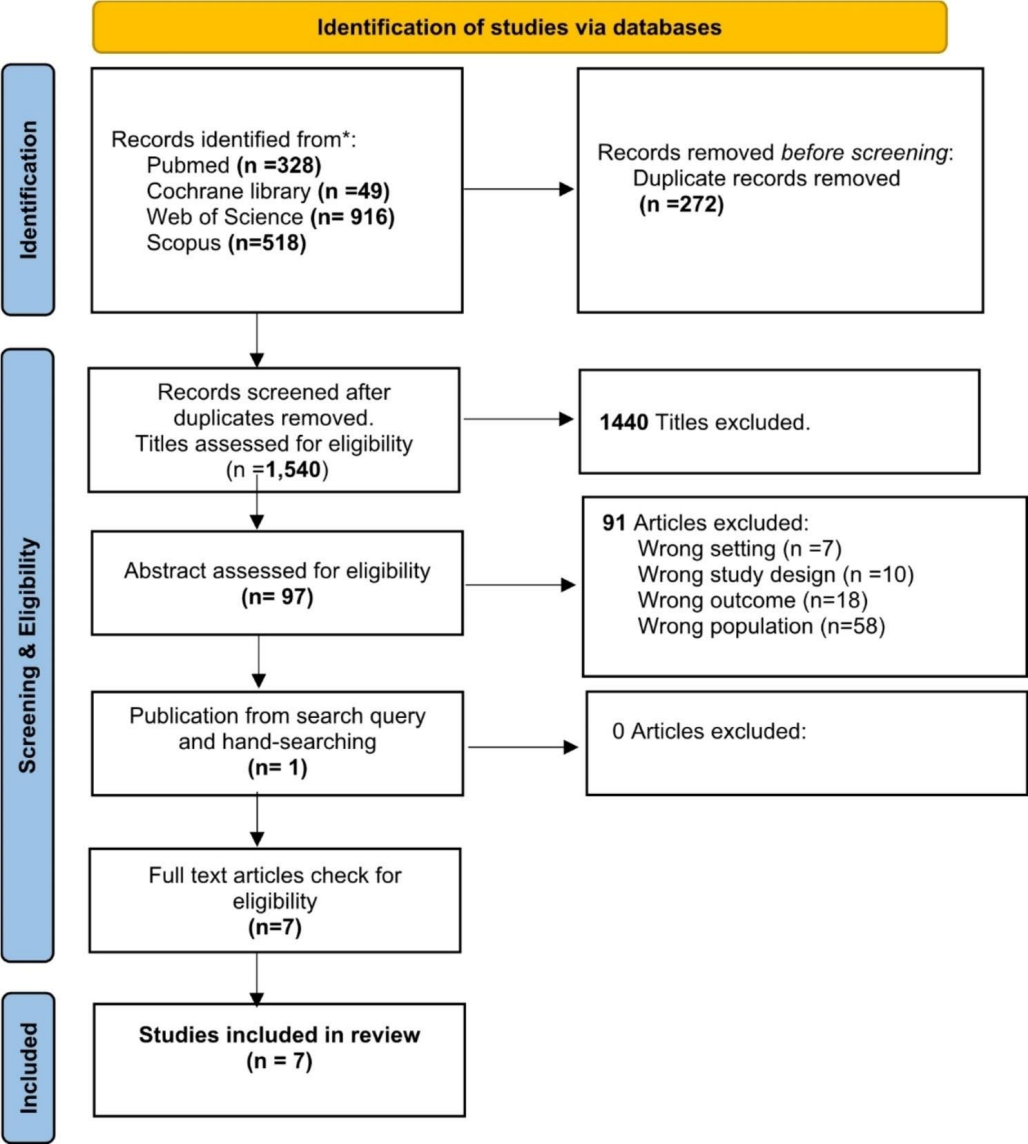
Flow chart of scoping review

A total of 1,811 records were identified through the electronic searches. Of these, 97 were deemed eligible and were assessed for study abstract. Articles were removed because they were case studies or did not include a sarcopenic obesity sample (n = 58), sarcopenic obesity was not the primary reported results of an intervention (n = 18), the articles were reviews or study design did not fit inclusion criteria (n = 10), and wrong intervention setting (n = 7). Additional one article was added from a hand searched method. Thus, a total of 7 trials were analysed in this scoping review. A flow diagram of the study selection procedure is shown in Fig. 1.

### Characteristics of included articles

Among the 7 included studies, none of the studies recruited both sexes, three studies recruited male subjects [36, 39, 40] while four studies recruited female as subjects [37, 38, 41, 42]. The sample size ranged from 16 to 139 subjects. Four out of six studies assessed the effectiveness of protein supplementation with combination of exercise training [36–39, 41]. One study evaluated the whole-body electromyostimulation and protein supplementation [40], while another study prescribed low calorie diet with protein supplementation to determine its effects on sarcopenic obesity [42]. Of these, six studies were randomised controlled trials [36–41] and one was a pre-post study design [42]. The majority (26%) of the studies was reported from Italy [38, 42] and German [39, 40], one from Canada [36], one from Japan [37], and one from Brazil [41]. The characteristics of the included studies have been summarized in Table 3.

**Table 3:** Characteristics & outcomes of included articles

Articles	Characteristics of participants (sample size, mean age)	Study design	Setting (Country)	Diagnosis algorithm	Interventions		Outcomes		
First author & Year					Mode of intervention	Content of Intervention	Effect on sarcopenia	Effect on obesity	Notable findings
Maltais et al., 2016 [31]	Total (N = 26); Control (n = 10), Intervention 1, Int 1 (n = 8), Int 2 (n = 8) Mean age = 65 ± 5 years old (y/o)	Randomised controlled trial (RCT), 4 months Control: Post-exercise shakes Intervention 1: Dairy Group Int 2: Non-dairy isocaloric and Isoprotein	Research center, Canada	Sarcopenia: appendicular lean mass index lower than 10.75 kg/m2 Obesity: BMI > 30 kg/m^2^	Three weekly 1 h-sessions, including a 10-min warm-up, were held on 3 non-consecutive days for 16 weeks. Drink the shake immediately after the exercise session Health education class: every 2 weeks	**Control**: Rice milk (0.6 g protein) 1.0-1.2 g protein/kg/day **Int 1**: Dairy shake (13.53 g protein, 7 g EAA) 1.3–2.1 g protein/kg/day **Int 2**: Non-dairy protein shake (12 g protein, 7 g EAA) 1.0-1.3 g protein/kg/day	Significant decreases were observed with Fat Mass only in the dairy supplement group. Resistance training significantly increased lean mass in all groups (p < 0.05) independently of supplementation (1.9 kg, nondairy shake; 1.7 kg, dairy shake; 1.4 kg, control).	Body weight significantly increased in the non-dairy shake group only (1.9 kg, p < 0.05). No changes were observed for body mass index.	Small number of participants were included in the group, statistical power to investigate any potential underlying mechanisms is limited. Resistance training combined with a milk-based post-exercise supplementation significantly reduced fat mass (FM) and increased lean mass (LM) No significant changes in biochemical profile (i.e. inflammatory marker). *men only
Kim et al., 2016 [32]	Total (N = 139); Control (n = 34), Int 1 (n = 36), Int 2 (n = 35) Int 3 (n = 34) Mean age= Control (81.1 ± 5.1 y/o), Int 1 (80.9 ± 4.2) Int 2 (81.4 ± 4.3) Int 3 (81.2 ± 4.9)	Randomised controlled trial (RCT), 3 months **Control**: Health education **Int 1**: Exercise and nutrition supplement, **Int 2**: Exercise **Int 3**: Nutritional supplement	Community based, Japan	Sarcopenic obesity : body fat percent of 32% or greater, measured by dual x-ray energy absorptiometry (DXA, Hologic QDR 4500 A), combined with skeletal muscle mass index less than 5.67 kg/m2 ;	Each exercise class was 60 min, twice per week Nutrition: Protein supplementation & Tea catechin was taken daily Edu class: every 2 weeks	**Protein supplementation**: 3 g leucine-enriched EAA **Tea catechin**: 540 mg catechin **Education class**: topic focused including cognitive function, long-term care insurance, etc. for elderly	Compared to control group, exercise (Ex) + nutrition (N) supplement group showed significant decreased in total body fat mass (p = 0.036) and increased stride (p = 0.038). Total body fat mass decreased in all intervention groups with greatest decrease found in Ex + N (5.5%, p = 0.036).	Exercise group showed decreased in trunk fat (p = 0.014).	The Ex + N and Ex interventions were over four times as likely to reduce body fat mass than the control group. Catechin can reduce body fat. Effects of exercise and nutrition alone were **insufficient in** **increasing muscle strength** among sarcopenic elderly people. No different in inflammatory biomarker. *women only
Sammarco et al., 2017 [33]	Total (N = 18); Control (n = 9), Intervention (n = 9), Mean age= Control (58 ± 10y/o), Intervention (53.9 ± 9)	Randomised controlled trial (RCT), 4 months **Control**: Hypocaloric diet + placebo **Int**: Hypocaloric + high protein diet	Primary care setting, Italy	Obesity diagnosed as fat mass > 34.8% and sarcopenia was defined when lean body mass was < 90% of the subject’s ideal fat free mass	Adherence to diet through a 7-day dietary record, dietitians followed up via phone calls every 2 weeks	**Control**: low calorie diet (minus 10% from REE calorimetry; 0.8-1 g protein/kg/day) **Intervention** 1: Low calorie + high protein diet (1.2–1.4 g protein/kg/day) with 15 g of protein of high biological value for each main meal	Women with high-protein diet preserved lean body mass and improved in muscle strength compared to control group.	Weight significantly decreased in both groups.	Dietary protein enrichment may represent a protection from the risk of sarcopenia following a hypocaloric diet (increased muscle strength score + 1.6 kg). *women only
Kemmler et al., 2017 [34]	Total (N = 100); Control (n = 34), Int 1 (n = 33), Int 2 (n-=33) Mean age= Control (76.9 ± 5.1y/o), Int 1 (77.1 ± 4.3), Int 2 (78.1 ± 5.1)	Randomised controlled trial (RCT), 4 months **Control**: Non-intervention **Int 1**: WB-EMS + protein supplementation Int 2: Isolated protein supplementation	Community based, German	Sarcopenia:EWGSOP Obesity: A percentage body fat ratio of > 27% (PBF) representing obesity	**WB-EMS**: 1.5 times per week, from 14 to 20 min after week 4 (4s electromuostimulation 4s rest) **Protein supplement**: Take with water, each time not more than 40 g (**no specific time on intake**)	**Protein supplement + Vit D**: Whey protein powder to achieve protein intake of 1.7-1.8 g/kg/day (per 100 g: 80 g whey protein, 9 g L-leucine, 57 g EAA)	Handgrip strength increased in the WB-EMS group (1.90 kg; P,0.001; P = 0.050 vs. control). Skeletal muscle mass increased significantly in both groups (P,0.001 and P = 0.043) and decreased significantly in the control group (p = 0.033).	Both intervention groups loss body fat (Int 1: 2.1%; Int 2: 1.1%), p < 0.001.	No adverse effects of WB-EMS or protein supplementation were found. *men only
Kemmler et al., 2018 [35]	Total (N = 100); Control (n = 34), Int 1 (n = 33), Int 2 (n-=33) Mean age= Control (76.9 ± 5.1y/o), Int 1 (77.1 ± 4.3), Int 2 (78.1 ± 5.1)	Randomised controlled trial (RCT), 4 months **Control**: Non-intervention **Int 1**: WB-EMS + protein supplementation **Int 2**: Isolated protein supplementation	Community based, German	Sarcopenia:EWGSOP Obesity: A percentage body fat ratio of > 27% (PBF) representing obesity	**WB-EMS**: 1.5 times per week, from 14 to 20 min after week 4 (4s electromuostimulation 4s rest) **Protein supplement**: Take with water, each time not more than 40 g (**no specific time on intake**)	**Protein supplement + Vit D**: Whey protein powder to achieve protein intake of 1.7-1.8 g/kg/day (per 100 g: 80 g whey protein, 9 g L-leucine, 57 g EAA)	No mention in this article.	Total body fat was reduced significantly in the protein group (− 3.6 ± 7.2%; p = 0.005) and WB-EMS + P (− 6.7 ± 6.2%; p < 0.001), but not in the control group (+ 1.6 ± 7.1%; p = 0.19). Trunk fat and waist circumference were decreased in intervention group (p < 0.001).	Moderate-high dosed of whey protein supplementation, especially when combined with WB-EMS, may be a feasible choice to address obesity and cardiometabolic risk in older sarcopenic obese adults. Treatment group showed improved in HDL-c but not statistic diff in TG and LDL-c level. *men only
Nabuco et al., 2019 [36]	Total (N = 26); Control (n = 13), Intervention (n = 13), Mean age= Control (70.1 ± 3.9 y/o), Intervention (68.0 ± 4.2)	Randomised controlled trial (RCT), 3 months Control: Placebo + supervised resistance training Intervention: Protein supplement + supervised resistance training	Community, Brazil	Sarcopenic: Appendicular lean soft tissue ALST < 15.02 kg Obesity: body fat mass ≥ 35%	**Protein**: Consumed only on training day **Resistance exercise**: (8 exercises, 3 × 8–12 rep, 3 times a week)	**Protein**: 35 g of whey protein (1.0 g protein/kg/day)	Intervention group presented greater increased in ALST (p < 0.05) compared to control group.	Intervention group showed decreased in trunk fat mass (p < 0.05) compared to control group.	Both groups showed improved (p < 0.05) scores for muscle strength, functional capacity, and metabolism biomarkers (IL-6) but no significant different between groups. Resistance training increased HDL-c, reduced glucose, TG, and CRP, without affecting LDL-c, insulin. *women only
Camajani et al., 2022 [37]	Total (N = 16); Mean age = 60 y/o (50–70 years)	Pre-post pilot study,45 days Intervention: Low calories diet + protein supplementation	Primary care, Italy	Sarcopenia: EWGSOP2 Obesity: Fat mass > 38%, according to NHANES III	**Low calories diet**: 1000 kcal/day (28% protein; 32% fat, 30% carbohydrate) **Protein**: taken at 5pm daily	**Protein**: 18 g whey protein (4.1 g of leucine); 5 mg vitamin D3; 1.38 g protein/kg/day	Women preserved total lean body mass and significantly improved their muscle strength, as measured by handgrip (15.3 vs. 20.1 Kg), and their muscle function.	A significant reduction in BMI (37.6 vs. 35.7 Kg/m2 ) and waist circumference (107 vs. 102.4 cm). Significant decreased in total trunk fat (p = 0.049).	No significant different in biomarkers. No significant adverse effects were recorded. **Significant increased in BUN, slight increased in** serum creatine and mild reduction in eGFR were found. *women only

*Whole-body electromyostimulation (WB-EMS)

*European Working Group on Sarcopenia in Older People (EWGSOP)

### Definition of sarcopenic obesity

In view of the definition of sarcopenia, different studies used different sarcopenia diagnoses. Skeletal muscle mass (Appendicular skeletal mass, ASM), lean body mass, ideal fat free mass, and skeletal muscle mass index were commonly used in most studies to determine sarcopenic condition [36–38, 41]. Three studies mentioned the used of the EWGSOP (European Working Group on Sarcopenia in Older People) as diagnostic criteria for sarcopenia in older adults [39, 40, 42]. In terms of obesity, the body fat percentage used was ranged between 27 and 38% [37–42]. Only one study that used body mass index (BMI) > 30 kg/m^2^ to determine the weight status of the subjects [36].

### Types of protein supplementation & protein intake

These studies included interventions of protein supplementation (Leucine enriched essential amino acid, EAA; whey protein) [36–42]. In one study, participants underwent a 3-month intervention involving the combination of catechin and a protein supplement [37]. The protein intake prescription by these studies ranged from 1.0 to 1.8 g/kg/BW/day for intervention group, for control group the protein intake was typically between 0.8 and 1.0 g/kg/BW/day [36, 38–42].

### Effects of different interventions on sarcopenic obesity

The effectiveness of the intervention in older adults was assessed through various sarcopenia evaluations. The most used sarcopenia measurement was lean muscle mass [36, 38] muscle strength [38, 42] handgrip strength [39, 42]. Measurement of body fat mass was seen in majority of the studies [36, 37, 39, 42]. In addition, body weight [36, 38], body trunk fat [36, 40, 42], and waist circumference [40, 42], were also measured to determine the effects on obesity.

The included intervention studies have consistently shown that exercise combined with protein supplement interventions, can lead to significant improvements in sarcopenic conditions such as muscle mass, strength, and physical function. Two studies utilised resistance training exercise in the intervention [36, 41], while one study combined both resistance exercise and aerobic exercise in the intervention [37]. The duration of exercise performed ranged from 2 to 3 times per week, with each session lasting 1 h [36, 37, 41]. However, one article did not report the duration of the exercise [36]. The exercise training also demonstrated a significant weight loss, loss of fat mass or trunk mass while preserving the lean muscle mass [36, 37, 41]. The results are the same as the intervention studies that utilised electromyostimulation (EMS) as an alternative to exercise [39]. Protein supplementation alone also showed improvement in sarcopenia measurements [38, 39, 42] as well as decrease in fat mass [36, 37], weight status [28], and waist circumference [42].

### Effects of interventions on metabolic and inflammatory biomarkers

Five studies examined the effects of intervention on metabolic and inflammatory biomarkers such as total cholesterol (CHOL), triglycerides (TG), low density lipoprotein, high density lipoprotein (HDL), C-reactive protein (CRP), and Interleukin-6 (IL-6) on the subjects [36, 37, 40–42]. Two studies reported no significant changes in cardiometabolic parameters and inflammatory biomarkers throughout the intervention period [36, 37, 42]. However, one study showed that EMS intervention improved the HDL and IL-6 level [40]. Another study showed that resistance training exercise increased the HDL level while the level of fasting glucose, TG, and CRP were decreased [41].

### Side effects of protein supplementation

No side effects have been reported in the clinical trials of included articles [36–41]. However, one study reported that there was some but not significant adverse effects on the subjects’ BUN, serum creatinine and eGFR with daily protein intake of 1.38 g/kg for 45 days [42].

### Discussion

The objective of this scoping review was to summarise the protein supplementation and exercise interventions that have demonstrated positive effectiveness in treating sarcopenic obesity (SO) among older adults. The results showed that protein supplementation alone (1.5 months to 4 months) improved the body weight, waist circumference, muscle strength and muscle mass [36–42]. Interventions that combined exercise and protein supplementation intervention demonstrated additional benefits, including improved inflammation markers, blood lipid profiles, fasting glucose levels, and greater impact on weight loss while preserving lean muscle mass in the sarcopenic obesity population [41]. By incorporating resistance exercise, which promotes muscle growth and strength [43], with whey protein supplementation, providing essential amino acids for muscle protein synthesis [28, 44], SO individuals have the potential to enhance muscle mass and function while managing their weight.

Numerous studies have examined the effects of whey and leucine protein supplementation as a nutritional intervention for sarcopenic obesity [37, 39–42]. Whey protein is a high-quality protein source that contains all the essential amino acids needed to support muscle growth and repair. Leucine, a branched-chain amino acid found in whey protein, plays a critical role in stimulating muscle protein synthesis. In combination, systematic review study has reported association between these protein supplementations and sarcopenia [45, 46]. Our study revealed whey and leucine supplementation (L-EAA) can increase muscle mass, improve muscle strength and function, and reduce body fat in individuals with sarcopenic obesity [37, 39–42]. On the other hand, protein supplement has been found to have a positive impact on metabolic health markers in combination with exercise training [36]. Unfortunately, only a small number of research have examined its impact on biomarkers in sarcopenic obesity-affected older adults.

The results of this scoping review are consistent with recent systematic reviews in older adults (50–70 years old) with sarcopenic obesity which have found that combining exercise with nutritional interventions provide advantages in reducing fat mass [33]. Most of the included studies were complimented with exercise intervention, this implies the importance of exercise training for sarcopenic obesity. Indeed, in line with previous studies, exercise interventions have demonstrated the ability to improve muscle mass, muscle strength, physical performance, and reduce fat mass [29, 32]. Resistance exercise can stimulate muscle hypertrophy due to training stimulus and lead to improvement in muscle strength and physical performance [47]. Resistance training exercise has been recognised as the primary treatment for sarcopenia in older adults, with a recommended frequency of two exercise sessions per week (1–3 sets of 6–12 repetitions) [48]. The recommended exercise prescription is largely consistent with our findings and previous research [33]. However, there is variation in the design of the exercise interventions, particularly in terms of the required sets, and the specific body parts targeted. [36, 37, 41], and no specific guidelines have been provided in this regard. On the other hand, combining resistance training with aerobic exercise may have potential benefits for sarcopenic obesity [37]. This approach has demonstrated improvements in ectopic fat deposition as well as physical and metabolic function among older adults with obesity [49].

In this review, we noticed some studies utilised the whole-body electromyostimulation (WB-EMS) as an alternative to exercise, [39, 41] which has shown to possibly improve muscle mass and strength among non-athletic adults [50]. Due to the limited number of studies that have utilised WB-EMS as an intervention, drawing conclusive findings based on the available evidence is challenging. Nonetheless, our results demonstrated that exercise interventions, particularly resistance exercise performed 2–3 times per week for 60 min per session, resulted in weight loss, reduction in body fat, trunk fat, and waist circumference. Considering the condition of obesity, further research is necessary to assess the exercise design, duration and types of exercise (i.e. incorporating aerobic exercise) necessary to improve the sarcopenic obesity as well as the efficacy of WB-EMS among sarcopenic obesity population.

This scoping review identified that three studies used the European Working Group on Sarcopenia in Older People (EWGSOP) for the definition of sarcopenia [39, 40, 42]. The EWGSOP recommends the use of a combination of different methods to diagnose sarcopenia based on the presence or absence of low muscle mass, low muscle strength, and/or low physical performance [51]. The other studies [36–38, 41] considered lean mass as diagnostic criteria for sarcopenia. This is in line with diagnostic criteria proposed by the European Society for Clinical Nutrition and Metabolism [52] and Society of Sarcopenia, Cachexia and Wasting Disorders [53] which considered lean muscle mass and gait speed are important predictors of mortality and physical disability in people with sarcopenia.

For obesity, different ranges of body fat percentage (27–38%) were used [37–42] except for one study [36] which used BMI as diagnostic criteria. The percentage of body fat index (PBF) has been considered as a more accurate standard than BMI to determine being overweight or obese because it measures body fat directly and BMI does not always reflect the true body fat in our body [54]. This distinction is particularly significant for sarcopenic elderly individuals, who often exhibit low muscle mass and high body fat while maintaining a seemingly normal BMI [13]. Consequently, it is crucial to utilize PBF (fat mass/total mass × 100) whenever possible when assessing the obesity status of sarcopenic older adults. Thus, by incorporating PBF measurements, healthcare professionals can obtain a more precise understanding of body composition and better identify and address obesity in this population.

The consensus guideline has recommended that for healthy older adults the protein intake should be at least 1.0 to 1.2 g of protein per kilogram of body weight (BW) per day [55, 56]. More protein is required (1.2–1.5 g/kg BW/d) for those who have acute or chronic diseases as suggested by PROT-AGE Study Group [55] and The Society of Sarcopenia, Cachexia and Wasting Disorders, which recommends the intake of 1.0 to 1.5 g/kg BW/day for older adults to maintain muscle mass [57]. This study showed the overall protein intake for intervention groups ranged between 1.0 and 1.8 g/kg BW/day which is slightly higher than 1.5 g/kg BW/day as recommended. One of the included articles reported some adverse effects on subjects’ renal profile with the daily protein intake of 1.38 g/kg BW during the intervention period [7]. Although the adverse effects of high protein intake did not appear to be significant, it is important to be cautious about recommending protein intake levels above 1.4 g per kilogram of body weight in older adults with sarcopenic obesity. In addition to protein, the role of micronutrients is also crucial for individuals with sarcopenic obesity. Insufficient intake of specific micronutrients, such as magnesium, selenium, calcium [58], vitamin B complex, vitamin D, and, iron [59] have been associated with the development of sarcopenia.

This scoping review has some limitations. The main shortcoming of this review was including studies that used different definitions for sarcopenic obesity which may reduce the comparability of results. However, the lack of a consensus on diagnostic criteria for sarcopenic obesity is an unavoidable challenge that needs to be addressed. Second, six of the seven included studies were conducted in Western countries, where body composition may differ from that of Asians and Caucasians. Therefore, the generalisability of the results might be limited. Third, the intervention periods of the included studies may be short to be representative of long-term effects. Studies on the role of protein supplements and exercise are of great public health importance and should be a priority.

### Conclusion

This scoping review provides an overview of nutritional and exercise approaches for managing sarcopenic obesity. Studies have demonstrated the effectiveness of exercise, specifically resistance exercise, and protein supplementation, such as whey protein, in addressing sarcopenic obesity in older adults, who experience age-related muscle mass and strength loss. However, exercise training may provide additional benefits beyond those provided by protein alone. In particular, exercise has been shown to have a positive impact on inflammatory markers, body weight, body fat trunk, and waist circumference. Therefore, a combination of resistance exercise (2–3 times/week) and protein supplementation may be the most effective approach for improving sarcopenic obesity and promoting healthy ageing. We suggest that a moderately high protein intake (1-1.3 g/kg BW/day) that take protein supplementation into account will be able to preserve muscle mass in individuals with sarcopenic obesity. Intake of more than (1.4 g/kg BW/day) should be prescribed with caution. Further research is needed to determine the optimal exercise and whether aerobic exercise should be incorporated for individuals with sarcopenic obesity.

## Data Availability

Not applicable.
